# Adherence to Diaphragm Use for Infection Prevention: A Prospective Study of Female Sex Workers in Kenya

**DOI:** 10.1155/2009/420196

**Published:** 2010-03-07

**Authors:** Elizabeth A. Bukusi, Maria F. Gallo, Anjali Sharma, Betty Njoroge, Denise J. Jamieson, Rosemary Nguti, April J. Bell, David A. Eschenbach

**Affiliations:** ^1^Center for Microbiology Research, Kenya Medical Research Institute, Mbagathi Rd., P.O. Box 54840-00200, Nairobi, Kenya; ^2^Department of Obstetrics and Gynecology, University of Nairobi, P.O. Box 30197, G.P.O, Nairobi, Kenya; ^3^Department of Global Health, University of Washington, 1959 N.E. Pacific St., Box 356355 Seattle, WA 98195-6355, USA; ^4^Department of Obstetrics and Gynecology, Department of Obstetrics & Gynecology, University of Washington, Box 356560, Seattle, WA 98195-6460, USA; ^5^Division of Reproductive Health, National Center for Chronic Disease Prevention and Health Promotion, Centers for Disease Control and Prevention, 4770 Buford Highway, Mail Stop K-34, Atlanta, GA 30341-3724, USA; ^6^International Training and Education Center on HIV (I-TECH), University of Washington, 901 Boren Ave., Suite 1100, Seattle, WA, 98104, UK; ^7^School of Mathematics, University of Nairobi, P.O. Box 30197, G.P.O, Nairobi, Kenya

## Abstract

*Objective*. To assess adherence to and acceptability of the diaphragm among 140 female sex workers in Kenya in a 6-month prospective study. *Methods*. At baseline and bimonthly visits, participants were interviewed on diaphragm knowledge, attitude, and practices. We used principal component analysis and logistic regression to identify predictors of consistent use. *Results*. At 50% of 386 bimonthly visits, women reported consistently using a diaphragm with all partners during the preceding 2 weeks. Consistent use was significantly higher at the 6-month than the 2-month visit. Women reported less covert use with “helping” (regular sex partners to whom she could go for help or support) than with “other” partners. Perceptions that diaphragms are easier to use than condoms and that their lack of coital interruption is important were associated with consistent diaphragm use with both partner types. Partner support of diaphragm use is correlated with consistent use with “helping” partners only while higher parity, consistent condom use, and perceived lack of need of condoms as a benefit of diaphragms were associated with consistent use with “other” partners. *Conclusions*. Diaphragm acceptance among female sex workers in Nairobi was high. Future studies should distinguish between partner types when evaluating diaphragm adherence.

## 1. Introduction

Cervical barriers such as diaphragms may protect women against the acquisition of sexually transmitted infections (STIs). Diaphragms could prevent *N. gonorrhoeae* and *C. trachomatis* from reaching the target columnar epithelial cells in the cervix [1] and protect against HIV acquisition by reducing the risk of the virus reaching HIV receptor-rich cells, which are present in larger numbers in the cervix than in the vagina [2–6]. In addition, diaphragms may reduce HIV susceptibility indirectly by protecting against other STIs [7, 8] and could be an effective mechanism with which to deliver microbicide in the genital tract if a microbicide effective against HIV were identified [9]. Although diaphragms have not been shown to be effective against HIV acquisition in a randomized controlled trial [10], observational data suggest that consistent diaphragm use could protect against STIs and their long-term sequelae [11]. 

Even if diaphragms were proven to be efficacious for STI prevention and safe for use in diverse populations, they would still have to be acceptable to potential users in order to be used consistently. Although diaphragm use has been shown to have a high rate of acceptability among women, many factors could influence adherence to diaphragm usage instructions, including the attributes of specific types of diaphragms (e.g., their reusability), how they are used (e.g., when they need to be inserted and removed, whether they are used with a microbicide gel, and whether users are instructed to refrain from concomitant douching or intravaginal cleansing), and partner knowledge of their use [12–21]. Although diaphragms are “female-controlled” devices, partners' attitudes toward their use could affect women's adherence to consistent usage. Covert diaphragm use, especially with regular partners, can be difficult to maintain long-term, and unintended discovery of diaphragm use could cause conflict between women and partners who object to the use of diaphragms [22, 23]. We conducted a 6-month prospective study to measure the prevalence of consistent diaphragm use and to identify determinants of consistent use among female sex workers in Kibera in Nairobi, Kenya.

## 2. Materials and Methods

### 2.1. Study Procedures

The study was conducted from 2004 to 2005 among female sex workers (i.e., women who reported trading sex for money or gifts in the prior 2 weeks), 18–57 years of age, in the Kibera settlement in Nairobi, Kenya who had previously participated in a randomized trial of monthly chemical prophylaxis for STI control and prevention [24]. Findings on the safety of diaphragm use and changes in condom use in this population will be reported separately. Women were ineligible for the study if they reported consistent condom use during the previous 2 months or if they had a current pregnancy, gynecological abnormalities precluding diaphragm use, latex allergies, a partner with latex allergies, or a history of toxic shock syndrome. 

At baseline, participants underwent a pelvic examination and were fitted for the silicone Milex Wide Seal diaphragm (Milex Inc., Chicago, IL). After being counseled on diaphragm use, women practiced inserting and removing their diaphragm under a clinician's observation. They were instructed to apply 1-2 teaspoons of K-Y Jelly (Johnson and Johnson; New Brunswick, New Jersey) to the cup and rim of the diaphragm for lubrication before inserting it and to keep the diaphragm in their vagina for at least 6 hours after coitus (without internal vaginal cleansing) but not for more than 24 hours. Women were given two diaphragms, a supply of K-Y Jelly, and male condoms.

At follow-up visits scheduled 1 week and 2, 4, 6 months after the baseline visit, women were counseled on safer sex practices and instructed to have their partners use a male condom during every coital act. They were counseled on the limited contraceptive effectiveness of diaphragms when used without spermicide and the unknown effectiveness of the diaphragm against STI acquisition. At all visits, they were tested for pregnancy and urinary tract infection and were treated syndromically for STIs and reproductive tract infections. The pelvic examination was repeated at the 6-month visit. 

Women were interviewed at the baseline visit and at the 2-, 4-, and 6-month follow-up visits to collect demographic data and data on their sexual behaviors; adherence to diaphragm use; and knowledge, attitude, and practices regarding diaphragms (as well as their perceptions of their partners' attitudes about diaphragm use). The questionnaires distinguished between diaphragm use with a “helping” partner and with all “other” partners. These partner types were established based on earlier formative research with the target population. A “helping” partner was defined as a regular sex partner to whom a woman could go for help or support if needed. If women had multiple partners in the past two weeks, they were asked to consider their main “helping” partner and their most recent “other” partner while answering the questionnaires. At all follow-up visits, participants completed a self-administered questionnaire about the frequency of their diaphragm, gel, and condom use for all acts with partners in the previous 2 weeks.

### 2.2. Statistical Analysis

SAS 9.1.3 (SAS Institute, Cary, NC) was used for data analysis. Consistent use was defined with a categorical variable that measured diaphragm use (with or without K-Y Jelly) for all coital acts in the previous 2 weeks and analyzed separately for “helping” and “other” partners. The analyses were restricted to follow-up visits in which women reported at least one coital act in the preceding 2-week period with the relevant partner type. The adjusted Wald method^24^ was used to calculate 95% confidence intervals for estimates of the percentage of women who used a diaphragm consistently, and chi-squared tests were used to assess differences between the percentages of women who reported consistent diaphragm use and covert diaphragm use at their 2- month visit and the percentages who did so at their 6-month visit.

In separate principal component analyses, we assessed the extent to which 25 and 26 factors were related to diaphragm use with “helping” partners and “other” partners, respectively. The association of each factor with diaphragm use was based on participants' responses to statements about the diaphragm, which originally were measured with a three-item Likert scale; however, we recoded the responses for our analysis as “agree” versus “neutral or disagree.” Principal component analysis is a method used to collapse a large number of partially correlated variables into fewer uncorrelated factors, each representing the combination of two or more of the initial variables [25]. This decreases the risk for type I errors (by reducing the number of statistical tests performed) as well as the risk for type II errors. Use of such a summary risk factor instead of multiple correlated items tends to produce stronger and more precise measures of association when a true association with the outcome of interest exists [26].

The derived variables then can be used in other analyses; for example, they can be regressed against the outcome of interest. We used the principal axis method to extract the components and the varimax method to produce orthogonal (uncorrelated) components. We based the decision to retain components on eigenvalues greater than 1 and the location of the break on the scree test. Factors that *load* for a given analysis are those that were given substantial weight when the principal component was constructed. We considered a factor to load for a component if the factor loading was at least .40 for the given component and less than .40 for the other components in the rotated factor pattern. The SAS procedure used for this analysis automatically standardized the component scores to unit variance. We retained two components in our subsequent analysis of factors related to diaphragm use with “helping” partners and with “other” partners. Tables 2 and 4 show the factors that loaded on each of the components. The communality estimate expresses the percentage of variance in the original variables that is accounted for by the retained principal components.

To identify determinants of consistent diaphragm use, we used logistic regression with generalized estimating equations, with the exchangeable working correlation matrix specified to adjust for correlation of standard errors between multiple records from one participant. After fitting individual models to perform bivariable analyses, we then fit a full model with all variables for the multivariable analysis. Using manual backward elimination, we removed factors that did not predict consistent diaphragm use based on an alpha of 0.05. Time-independent factors that were assessed as predictors of consistent diaphragm use were measured at baseline; these factors included age (≤27, 28–34, ≥35 years), marital status (never married and not cohabiting versus cohabiting, divorced, or widowed), highest educational level completed (<8 versus 9–12 years), ethnicity (Kikuyu versus other), weekly income (≤9 versus >9 USD), parity (0–1 versus ≥2 children), perceived importance of preventing pregnancy (not at all versus moderately or a lot), worry about pregnancy (not at all versus moderately or a lot), and worry about HIV (not at all versus moderately or a lot). Time-dependent factors describing behaviors in the preceding 2 weeks were measured at the bimonthly visits, these included number of sex partners (1–5 versus ≥6), number of new sex partners (0, 1–2, ≥3), coital frequency with all partners (0–5, 6–15, ≥16 acts), any condom use with “helping” or “other” partners (yes versus no), a variable for the bimonthly (2, 4, or 6-month) visit, the components constructed in the principal component analysis; and the variables that did not load on the components created in the principal component analysis.

Only women who gave written, informed consent participated. Ethical review committees at the University of Nairobi, the University of Washington, the University of California, San Francisco, and the US Centers for Disease Control and Prevention approved the research.

## 3. Results

### 3.1. Study Population

Of the 180 women who were screened, 140 met the eligibility criteria and were enrolled in the study. Most women completed the 2-month (*n* = 134), 4-month (*n* = 130), and 6-month visits (*n* = 126). Overall, 138 (99%) of women enrolled in the study completed at least one of the bimonthly visits, and 121 (86%) completed all three bimonthly visits. Study attrition was due to pregnancy (*n* = 8), moving (*n* = 3), and loss to follow-up (*n* = 3). Analyses of diaphragm use with “helping” partners were based on data collected during 313 bimonthly visits by 121 women who reported coitus with a “helping” partner during at least one bimonthly visit. Analyses of diaphragm use with “other” partners were based on data collected during 362 bimonthly visits by 135 women who reported at least one act with any “other” type of partner during at least one bimonthly visit. Analyses of diaphragm use with all partners were based on data collected during 386 bimonthly visits by 138 women who reported at least one act with either partner type during at least one bimonthly visit. Participants' median age was 30 years (range, 18–55); 73% were cohabiting, divorced, or widowed; 81% had less than 8 years of education (Table 1). Most participants (63%) reported having previously used condoms for contraception. Only one woman reported having ever used a diaphragm before. 

### 3.2. Adherence and Covert Use

Women reported that they used a diaphragm with all partners during the preceding 2 weeks at 50% of the bimonthly *visits*, with all “helping” partners at 59% of the *visits*, and with all “other” partners at 64% of the* visits*. Among the subset reporting at least one act with the relevant partner type during the bimonthly visits, 41%, 45%, and 33% of *women *reported consistent diaphragm use with “helping,” “other,” or all partners, respectively.

**Figure 1:**
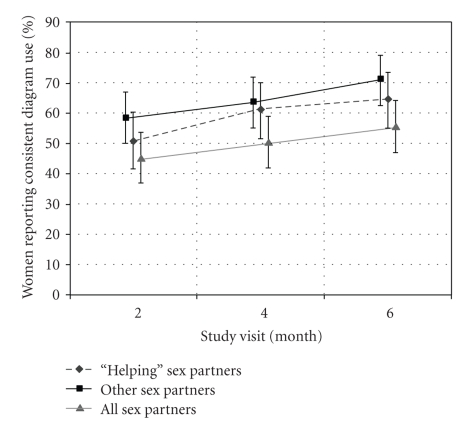
Prevalence of consistent diaphragm use, by partner type and study visit, Diaphragm Acceptability Study, Nairobi, Kenya, 2004-2005. Consistent use defined as diaphragm use during 100% of coital acts in the previous 2 weeks. Analysis restricted to women with at least one coital act in the previous 2 weeks with the relevant partner type. Bars represent 95% confidence intervals.

The percentage of women who reported consistent diaphragm use increased over the course of the study (Figure 1). The proportion of women who reported consistent diaphragm use was significantly higher at the 6-month visit than the 2-month visit for coital acts with “helping” partners (*P* = .04) and with “other” partners (*P* = .04) but not for coital acts with all partners (*P* = .09). 

Results of analyses limited to visits by women who reported at least some diaphragm use with the relevant partner type showed that women reported always using the diaphragm without their “helping” partners' knowledge at 55% of the bimonthly visits and without their “other” partners' knowledge at 76% of the visits. The percentage of women who reported consistent covert use of the diaphragm during the preceding 2 weeks did not vary significantly between the 2- and 6-month visits either with “helping” partners (58% and 51%, resp.; *P* = .56) or with “other” partners (77% and 73%, resp.; *P* = .82).

### 3.3. Diaphragm Use with “Helping” Partners

Principal component analysis yielded two components associated with the use of a diaphragm with a “helping” partner (Table 2). The first component was participants' perception of the degree to which their “helping” partner would support their diaphragm use. Among the factors that loaded on this component was a woman's agreement that diaphragm use would be unlikely to cause her “helping” partner to refuse sex, argue about sex, end their relationship, stop supporting her, or become mad at her. The second component was a composite of woman's attitudes toward four attributes of diaphragms: liking the extra lubrication and viewing lack of hormonal side effects, lubrication, and ability to have uninterrupted sex as benefits of product use.

Results from the unadjusted analysis showed that five variables were associated with reporting consistent diaphragm use with a “helping” partner in the prior 2 weeks: the time of the follow-up visit (6-month versus 2-month visit), perceived partner support of diaphragm use, attitudes toward diaphragm attributes, the perception that using a diaphragm with gel use is at least as easy as using a condom, and the perceived importance of diaphragm use not interrupting sex (Table 3). In the multivariable analysis, however, the associations remained significant for only three variables: perceived partner support of diaphragm use (adjusted odds ratio [OR], 1.4; 95% confidence interval [CI], 1.1–1.7), the perception that using a diaphragm with gel use is at least as easy as using a condom (adjusted OR, 2.0; 95% CI, 1.2–3.1), and the perceived importance of diaphragm use not interrupting sex (adjusted OR, 2.8; 95% CI, 1.1–7.1).

### 3.4. Diaphragm Use with “Other” Partners

Results from the principal component analysis showed two components significantly associated with diaphragm use with “other” partners (Table 4). The first was a composite of factors concerning women's perceptions of whether their “other” partners supported their diaphragm use, including whether diaphragm use would cause their “other” partners to become mad, angry, argue with her, refuse to have sex, or hit or beat her. The second component was based on the same four factors as the second component in the analysis of diaphragm use with “helping” partners.

Nine variables were related to consistent diaphragm use with “other” partners in the previous 2 weeks (Table 5). Four of these variables, however, were not associated with consistent diaphragm use in the adjusted model: study follow-up visit (6-month versus 2-month), number of recent sex partners, number of coital acts with all partners in the previous 2 weeks, and the second component. Only the following five variables were associated with consistent diaphragm use with “other” partners in the multivariable model: having ≥2 children (adjusted OR, 2.1; 95% CI, 1.1–4.0), using condoms consistently (adjusted OR, 2.1; 95% CI, 1.2–3.7), perceiving diaphragm use to be easier than condom use (adjusted OR, 2.3; 95% CI, 1.4–3.8), perceiving diaphragm use not interrupting sex as being important (adjusted OR, 2.2; 95% CI, 1.0–5.0), and perceiving the lack of need for condoms as a benefit of diaphragm + gel use (adjusted OR, 2.3; 95% CI, 1.3–4.2).

### 3.5. Reasons for Not Using a Diaphragm

The most common explanation that women gave for not using a diaphragm was that they used condoms instead; 75% of the women who reported inconsistent diaphragm use during the previous 2 weeks cited this reason one or more times (Table 6). This explanation was provided more often to explain not using a diaphragm with “other” partners (82%) than with “helping” partners (38%). Women often cited “trust” and “knowing partners well enough not to use it” as reasons for not using diaphragms with “helping” partners but rarely given cited either as a reason for not using a diaphragm with “other” partners. Another frequently cited reason for not using a diaphragm was that sex was unexpected or that women did not have the diaphragm with them (cited by 30% as a reason with “helping” partners and by 53% as reasons with “other” partners). In contrast, negative attributes of the diaphragm (e.g., participant discomfort) were rarely reported as reasons for not using it.

## 4. Discussion

A substantial proportion of participants reported consistent diaphragm use in the prior 2 weeks with “helping” and “other” partners (59% and 64% of follow-up visits, resp.). Diaphragm use with each partner type increased over the course of the study. With one exception [12], results from studies of diaphragm use for infection control have shown little change over time in the percentage of women who reported consistent use during study visits [15, 17, 19, 21]. The increase that we found in the percentage of women who used diaphragms consistently might be the product of a “learning curve,” whereby women learned to use the device more consistently or became more comfortable with its use over time. Because of the low attrition rate in the study, the increase in the rate of consistent diaphragm use is unlikely to be attributable completely to early study discontinuation by women who did not like the diaphragm.

The perceptions that diaphragms are easier to use than condoms and that lack of interruption to sex is important were associated with consistent diaphragm use with both partner types. The association of other factors with consistent diaphragm use, however, differed by partner type. Women's perception of the degree to which their “helping” partner would support diaphragm use (measured by the principal component analysis) was associated with recent consistent diaphragm use with this partner but not with “other” partners. This difference by partner type could be related to a higher rate of awareness of diaphragm use among “helping” than among “other” partners. Women might have felt a responsibility to inform “helping” partners about their diaphragm use or have judged covert use to be difficult to sustain long term with this partner. They also might have been more concerned about the consequences of a “helping” partner discovering their covert diaphragm use. On the other hand, condom-related factors (reporting consistent condom use and perceiving the lack of need for condoms as a benefit of diaphragm use) were associated with consistent diaphragm use with “other” partners but not with “helping” partners. Overall, our results suggest that future studies of the effect of diaphragm promotion on condom use should account for partner type.

The study's main limitation was its reliance on self-reported data, which has potential for reporting bias. For example, we cannot eliminate the possibility that the apparent increase in diaphragm use over the course of the study resulted from a change in the validity of self-reporting. Women might have overreported consistent diaphragm use more often as the study progressed if they developed closer rapport with the study staff and, thus, had a stronger motivation to “please” staff by reporting adherence to their instructions. Similarly, the association between consistent condom use and consistent diaphragm use with “other” partners could be spurious if the perceived social desirability of using both products caused some women to overreport their use of them. Another limitation is that because the study population consisted of female sex workers in Kibera, Nairobi who did not consistently use condoms and who were willing to try the diaphragm, study findings might not be generalizable to other populations. Finally, just as intention to use study products does not necessarily predict use [27, 28], the level of adherence to consistent diaphragm use (as well as factors related to adherence) among participants in this study might be different in nonstudy settings or be impossible to sustain for periods longer than 6 months.

Although study participants were advised repeatedly of the unknown effectiveness of the diaphragm against STI acquisition and, when used for this purpose, against pregnancy, qualitative data from the exit interview (data not reported here) suggest that women were reluctant to return the diaphragm at the end of the study because they believed that it protected them from acquiring STIs. This finding has been observed elsewhere [14] and highlights the need for better methods for ensuring participant comprehension of key points during research participation. The quantitative measures of participant concern about HIV acquisition (as well as concern about pregnancy and the perceived importance of preventing pregnancy) were not associated with consistent diaphragm use. 

Reasons cited for not using the diaphragm with “other” partners included having unexpected sex or not having the diaphragm available. Continuous diaphragm use (except during daily removal for cleaning) could address this barrier. By making its use independent of coitus, continuous use of the diaphragm could increase the proportion of sex acts protected by the device and thus improve its effectiveness [29]. However, little research has been conducted on continuous diaphragm use [30], and the safety and effectiveness of such use would need to be proven in future studies before continuous use of diaphragms could be promoted.

Other research has demonstrated that relationship factors influence diaphragm use [31]. To our knowledge, though, this was the first prospective study to evaluate adherence to diaphragm use by partner type. We used only two classifications for partner type; a more refined understanding of partners might have yielded different results. Nevertheless, the differences we found in determinants of consistent diaphragm use during sex with “helping” partners and during sex with “other” partners suggest that future research should also distinguish between different types of partners when evaluating adherence to diaphragm use. Tailoring interventions and counseling messages based on women's types of partners could perhaps help improve efforts to promote consistent diaphragm use.

## Figures and Tables

**Table 1:** Baseline sociodemographic and selected characteristics among study participants who completed at least one follow-up visit (*N* = 138), Nairobi, Kenya, 2004-2005.

Characteristics	No.	(%)
Age		
≤27 years	47	(34)
28–34 years	46	(33)
≥35 years	45	(33)
Marital status		
Never married and not cohabiting	37	(27)
Cohabiting, divorced or widowed	101	(73)
Ethnicity		
Kikuyu	68	(49)
Other	70	(51)
Education completed		
0–8 years	112	(81)
9–12 years	26	(19)
Income per week		
≤9 US dollars	70	(51)
>9 US dollars	68	(49)
Current primary birth control method		
None	19	(14)
Condoms	49	(40)
Hormonal contraception	47	(38)
IUD or tubal ligation	8	(7)
	Median	(Range)

Number of children	3	(0–10)
Age at first intercourse	16	(10–25)
Vaginal cleansing in past week	11	(1–30)
Sexual partners in past two weeks	5	(1–56)

**Table 2:** Rotated factor pattern and final communality estimates from principal component analysis of participants' (and participants' perception of partners') knowledge, attitude, or practices regarding diaphragm use with “helping” partner, Nairobi, Kenya, 2004-2005*.

Knowledge, attitude, or practices^†^	Component 1 (Perceived partner support of diaphragm use)*	Component 2 (Attitudes toward study product attributes)*	Communality estimate
Unlikely that “helping” partner would make fun of participant for diaphragm + gel use	.58*	.01	.33
Unlikely that “helping” partner would argue because of diaphragm + gel use	.89*	.12	.80
Unlikely that “helping” partner would get mad because of diaphragm + gel use	.87*	.14	.77
Unlikely that “helping” partner would hit or beat participant because of diaphragm + gel use	.76*	.05	.57
Unlikely that “helping” partner would refuse sex because of diaphragm + gel use	.91*	.13	.84
Unlikely that “helping” partner would end relationship because of diaphragm + gel use	.89*	.12	.81
Unlikely that “helping” partner would stop support because of diaphragm + gel use	.89*	.08	.80
If participant wanted to use diaphragm + gel and “helping” partner did not, he would not refuse to have sex with diaphragm + gel	.69*	.06	.48
If participant wanted to use diaphragm + gel and “helping” partner did not, he would not talk participant out of use	.51*	.14	.28
If participant wanted to use diaphragm + gel and “helping” partner did not, he would not insist on non-use	.51*	.13	.28
“Helping partner” likes extra lubrication from diaphragm + gel use	.46*	.31	.30
Likes extra lubricant from diaphragm + gel use	.10	.63*	.41
No hormonal side effects is benefit of diaphragm + gel use	.01	.51*	.26
Lubrication is benefit of diaphragm + gel use	.09	.63*	.40
Uninterrupted sex is benefit of diaphragm + gel use	.10	.62*	.39

*Factor loading was .40 or greater for the given component; based on 313 intervals from 121 women who reported having sex with “helping” partner during at least one follow-up interval.

^†^ *The following factors did not load on either component: Diaphragm* + *gel use is easier than condom use; Diaphragms* + *gel are very effective in protecting against HIV or other STDs; Diaphragms* + *gel are very effective in protecting against pregnancy; Important to use diaphragms* + *gel with all partners; Does not prefer condoms to diaphragm* + *gel; Plans to use diaphragm* + *gel at next coitus with "helping" partner; Ability to use without partner permission is benefit of diaphragm* + *gel use; Ability to use without partner awareness is benefit of diaphragm* + *gel use; No need for condoms is benefit of diaphragm* + *gel; Important that diaphragm use does not interrupt sex.*

**Table 3:** Associations between consistent diaphragm use with “helping” partner during previous 2 weeks and selected demographic characteristics and attitudes toward diaphragm use, Nairobi, Kenya, 2004-2005*.

	No. of intervals with consistent use	No. of intervals without consistent use	Bivariable model		Multivariable model^†^	
			OR	(95% CI)	OR	(95% CI)
*Time-independent factors*						
Age						
≤27 years	63	49	Referent			
28–34 years	75	45	1.4	(0.7, 2.7)		
≥35 years	46	35	1.1	(0.5, 2.4)		

Marital status						
Never married and cohabiting	57	34	1.2	(0.6, 2.2)		
Cohabiting, divorced or widowed	127	95	Referent			

Education completed						
0–8 years	141	104	Referent			
9–12 years	43	25	1.3	(0.6, 2.8)		

Parity						
0-1 children	45	35	Referent			
≥2 children	139	94	1.2	(0.6, 2.3)		

*Time-dependent factors*						
Study follow-up visit						
2-month	55	53	Referent			
4-month	63	40	1.4	(0.9, 2.2)		
6-month	66	36	1.5	(1.0, 2.4)		

All sex partners in past 2 weeks						
1–5	106	61	1.2	(0.8, 1.9)		
6–42	78	68	Reference			

Coital acts with all partners in past 2 weeks						
0–5 acts	31	17	1.5	(0.7, 2.8)		
6–15 acts	109	73	1.3	(0.8, 2.2)		
≥16 acts	44	39	Referent			

Consistent condom use with “helping” partner in past 2 weeks						
Yes	57	26	1.5	(0.9, 2.6)		
No	127	103	Referent			

Component 1 (Perceived partner support of diaphragm use)			1.3	(1.1, 1.7)	1.4	(1.1, 1.7)
Component 2 (Attitudes toward study product attributes)			1.2	(1.0, 1.5)		

Diaphragm + gel use is easier than condom use						
Yes	140	81	1.9	(1.2, 3.1)	2.0	(1.2, 3.1)
No	44	48				

Important to participant that diaphragm use does not interrupt sex						
Yes	176	119	2.7	(1.1, 6.6)	2.8	(1.1, 7.1)
No	8	10	Referent			

*OR = odds ratio; CI = confidence interval*

*Analyzed with logistic regression model with generalized estimating equations based on 313 intervals from 121 women who reported having sex with “helping” partner during at least one follow-up interval.

^†^ *Adjusted for all variables in column.*

*The following variables also were analyzed but were not associated with consistent diaphragm use: ethnicity (Kikuyu versus other), education (*≤*9 USD versus *>*9 USD); important to prevent pregnancy (not at all versus moderately or a lot); worry about pregnancy (not at all versus moderately or a lot); worry about HIV (not at all versus moderately or a lot); new main sex partners in past 2 weeks (0 versus 1-2 versus *≥*3); under the influence of alcohol during sex with “helping” partner in past 2 weeks (never versus *≥*1 time); under the influence of “bhang” or other drugs during sex with “helping” partner in past 2 weeks (never versus *≥*1 time); and the remaining factors that did not load in principal component analysis (listed in the footnote for Table 2).*

**Table 4:** Rotated factor pattern and final communality estimates from principal component analysis of participants' (and perception of partners') knowledge, attitude, or practices regarding diaphragm use with “other” partners, Nairobi, Kenya, 2004-2005*.

Knowledge, attitude, or practices^†^	Component 1 (Perceived partner support of diaphragm use)*	Component 2 (Attitudes toward study product attributes)*	Communality estimate
Unlikely that “other” partners would make fun of participant for diaphragm + gel use	.64*	.39	.57
Unlikely that “other” partners would get angry because of diaphragm + gel use	.86*	.31	.83
Unlikely that “other” partners would argue because of diaphragm + gel use	.86*	.27	.81
Unlikely that “other” partners would get mad because of diaphragm + gel use	.89*	.28	.86
Unlikely that “other” partners would hit or beat her because of diaphragm + gel use	.72*	.31	.62
Unlikely that “other” partners would refuse sex because of diaphragm + gel use	.83*	.28	.76
If participant wanted to use diaphragm + gel and “other” partners did not, he would not refuse to have sex with diaphragm + gel	.67*	.19	.48
If participant wanted to use diaphragm + gel and “other” partners did not, he would not talk participant out of use	.52*	.27	.34
If participant wanted to use diaphragm + gel and “other” partners did not, he would not refuse to pay	.61*	.21	.41
If participant wanted to use diaphragm + gel and “other” partners did not, he would still have sex with you	.66*	.26	.49
If participant wanted to use diaphragm + gel and “other” partner did not, he would not insist on nonuse	.58*	.30	.42
Likes extra lubrication from diaphragm + gel use	.09	.55*	.31
No hormonal side effects is benefit of diaphragm + gel use	.10	.50*	.26
Lubrication is benefit of diaphragm + gel use	.07	.56*	.32
Uninterrupted sex is benefit of diaphragm + gel use	.10	.61*	.39

*Factor loading was .40 or greater for the given component; based on 362 bimonthly study visits from 135 women who reported having sex with “other” partner during at least one bimonthly visit.

^†^ *The following factors did not load on either component: “Other” partner likes extra lubrication from diaphragm* + *gel use; Diaphragm* + *gel use is easier than condom use; Diaphragms* + *gel are very effective in protecting against HIV or other STDs; Diaphragms* + *gel are very effective in protecting against pregnancy; Important to use diaphragms* + *gel with all partners; Does not prefer condoms to diaphragm* + *gel; Plans to use diaphragm* + *gel at next coitus with "other" partner; Ability to use without partner permission is benefit of diaphragm* + *gel use; Ability to use without partner awareness is benefit of diaphragm* + *gel use; No need for condoms is benefit of diaphragm* + *gel; Important that diaphragm use does not interrupt sex.*

**Table 5:** Associations between consistent diaphragm use with “other” partners in previous 2 weeks and selected demographic characteristics and attitudes toward diaphragm use, Nairobi, Kenya, 2004-2005*.

	No. of intervals with consistent use	No. of intervals without consistent use	OR	(95% CI)	OR	(95% CI)
			Bivariable model		Multivariable model^†^	
*Time-independent factors*						
Age						
≤27 years	73	53	Referent			
28–34 years	81	40	1.5	(0.8, 3.0)		
≥35 years	79	36	1.6	(0.8, 3.3)		

Marital status						
Never married and cohabiting	64	36	Referent			
Cohabiting, divorced or widowed	169	93	1.1	(0.6, 2.0)		

Education completed						
0–8 years	193	105	1.1	(0.5, 2.2)		
9–12 years	40	24	Referent			

Parity						
0–1 children	45	43	Referent		Referent	
≥2 children	188	86	2.2	(1.2, 4.0)	2.1	(1.1, 4.0)

*Time-dependent factors*						
Study follow-up visit						
2-month	75	53	Referent			
4-month	78	44	1.2	(0.8, 1.8)		
6-month	80	32	1.7	(1.1, 2.6)		

All sex partners in past 2 weeks						
1–5	116	54	1.5	(1.0, 2.1)		
6–42	117	75	Referent			

Coital acts with all partners in past 2 weeks						
0–5 acts	41	14	2.1	(1.0, 4.3)		
6–15 acts	129	72	1.2	(0.8, 2.0)		
≥16 acts	63	42	Referent			

Consistent condom use with “other” partners in past 2 weeks						
Yes	165	70	2.0	(1.2, 3.4)	2.1	(1.2, 3.7)
No	68	59	Referent		Referent	
Component 1 (Perceived partner support of diaphragm use)			1.2	(0.9, 1.4)		
Component 2 (Attitudes toward study product attributes)			1.2	(1.0, 1.5)		

Diaphragm + gel use is easier than condom use						
Yes	189	79	2.5	(1.5, 4.1)	2.3	(1.4, 3.8)
No	44	50	Referent		Referent	

Important that diaphragm use does not interrupt sex						
Yes	222	117	2.9	(1.3, 6.5)	2.2	(1.0, 5.0)
No	11	12	Referent			

No need for condoms is benefit of diaphragm + gel use						
Yes	50	26	1.8	(1.0, 3.1)	2.3	(1.3, 4.2)
No	183	103	Referent		Referent	

*OR = odds ratio; CI = confidence interval.*

*Analyzed with logistic regression model with generalized estimating equations based on 362 bimonthly study visits from 135 women who reported having sex with “other” partner during at least one bimonthly visit.

^†^ *Adjusted for all variables in column.*

*The following variables also were analyzed but were not associated with consistent diaphragm use: ethnicity (Kikuyu versus other), education (*≤*9 USD versus *>*9 USD), important to prevent pregnancy (not at all versus moderately or a lot); worry about pregnancy (not at all versus moderately or a lot); worry about HIV (not at all versus moderately or a lot); new sex partners in past 2 weeks (0 versus 1-2 versus *≥*3); and the remaining factors that did not load in principal component analysis (listed in the footnote for Table 4).*

**Table 6:** Reasons cited for not using diaphragm in previous 2 weeks, overall and by partner type, Nairobi, Kenya, 2004-2005*.

			Partner type			
	Overall (*N* = 92)		“Helping” (*N* = 71)		“Other” (*N* = 74)	
Reasons for not using condoms	No.	(%)	No.	(%)	No.	(%)
*Reasons related to partner or coital act*						
Afraid to ask	6	(7)	4	(6)	2	(3)
Did not need protection from STD	1	(1)	0	(0)	1	(1)
Trust each other	44	(48)	43	(61)	2	(3)
Know each other well enough not to use it	43	(47)	43	(61)	4	(5)
Used condoms instead	69	(75)	27	(38)	61	(82)
Diaphragm uncomfortable for partner	8	(9)	8	(11)	0	(0)
Partner objected to use	28	(30)	20	(28)	11	(15)
Unexpected sex/did not have diaphragm	46	(50)	21	(30)	39	(53)
Could not use because drunk	16	(17)	9	(13)	11	(15)
Forgot to use diaphragm	28	(30)	14	(20)	17	(23)
Lost diaphragm	2	(2)	2	(3)	2	(3)
*Device-related reasons*						
Diaphragm uncomfortable for participant	3	(3)	1	(1)	2	(3)
Do not like having genitals touched	3	(3)	3	(4)	0	(0)
Difficulties inserting or removing	0	(0)	0	(0)	0	(0)
Did not need pregnancy protection	3	(3)	2	(3)	1	(1)
Do not think effective against STD	1	(1)	1	(1)	0	(0)

*Barriers reported during at least one bimonthly follow-up visit. Participant could give multiple reasons. Analysis restricted to the subset of participants reporting inconsistent diaphragm use in the past two weeks with the relevant partner type during at least one bimonthly visit.
